# I-TREAT: Internet-based Cognitive Behavioral Treatment for Other Specified Feeding or Eating Disorders (OSFED) in Danish Adolescents and Adults – Study Protocol for a Randomized Controlled Trial

**DOI:** 10.1192/j.eurpsy.2023.1112

**Published:** 2023-07-19

**Authors:** E. Runge, M. B. Lichtenstein

**Affiliations:** Research Unit for Digital Psychiatry, Center for Digital Psychiatry, Mental Health Services in the Region of Southern Denmark, Odense C, Denmark

## Abstract

**Introduction:**

Eating disorders severely impair psychosocial functioning, physical health, and quality of life. In particular, Anorexia Nervosa has the highest mortality rate among all psychiatric diseases. Other Specified Feeding or Eating Disorders have the highest lifetime prevalence with weighted means of 7.64% for women. Eating disorders are considered hard-to-treat, and studies have indicated that people suffering from eating disorders prefer low-threshold interventions compared to traditional mental health care. International studies show promising results of internet-based interventions for Other Specified Feeding or Eating Disorders.

**Objectives:**

To test the effectiveness of an internet-based psychologist-guided cognitive behavioral treatment program (I-TREAT) to reduce eating disorder symptoms in Danish adolescents and adults with Other Specified Feeding or Eating Disorders.

**Methods:**

This study is a randomized controlled trial with two arms: 1) an intervention group and 2) an active control group. Participants are adolescents from the age of 15 or above, as well as adults, diagnosed with Other Specified Feeding or Eating Disorders. The intervention group receives the I-TREAT program while the control group receives self-guided mindfulness exercises. I-TREAT comprises 12 text-based treatment sessions with psychoeducation and treatment-related tasks, based on cognitive behavioral therapy and elements of compassion-focused therapy. The treatment duration is approximately 12 to 36 weeks. Videos, pictures, and animations support the treatment content and the program is accessible by web-browser and app. A specialist in eating disorders guides the patient through treatment with task-related feedback and asynchronous written communication. Patients will answer questionnaires regarding eating disorder symptoms, quality of life, and motivational states before, during, and after treatment, with follow-up measures at 3, 6, and 12 months. We expect to include 63 patients to each group and commence recruitment in August 2023. Preliminary results from a feasibility study on I-TREAT show good evaluations from clinicians and patients (N=30).

**Results:**

No results have yet been obtained. The results will be submitted to international scientific journals and presented at conferences.

**Conclusions:**

The internet-based cognitive behavioral treatment program I-TREAT may be a promising tool for effectively treating adolescents and adults with Other Specified Feeding or Eating Disorders in Denmark.

**Disclosure of Interest:**

None Declared

